# yRACK1/Asc1 proxiOMICs—Towards Illuminating Ships Passing in the Night

**DOI:** 10.3390/cells8111384

**Published:** 2019-11-04

**Authors:** Kerstin Schmitt, Oliver Valerius

**Affiliations:** Department of Molecular Microbiology and Genetics, Institute of Microbiology and Genetics, Göttingen Center for Molecular Biosciences (GZMB), Georg-August-University Göttingen, 37077 Göttingen, Germany; kschmit1@gwdg.de

**Keywords:** Asc1/RACK1, Rps2, Rps3, Ubp3-Bre5, Def1, Vps30, biotin identification, ribosome, translation, ribosome-associated quality control (RQC), autophagy, mRNA-binding protein

## Abstract

Diverse signals and stress factors regulate the activity and homeostasis of ribosomes in all cells. The *Saccharomyces cerevisiae* protein Asc1/yRACK1 occupies an exposed site at the head region of the 40S ribosomal subunit (*hr40S*) and represents a central hub for signaling pathways. Asc1 strongly affects protein phosphorylation and is involved in quality control pathways induced by translation elongation arrest. Therefore, it is important to understand the dynamics of protein formations in the Asc1 microenvironment at the *hr40S*. We made use of the in vivo protein-proximity labeling technique Biotin IDentification (BioID). Unbiased proxiOMICs from two adjacent perspectives identified nucleocytoplasmic shuttling mRNA-binding proteins, the deubiquitinase complex Ubp3-Bre5, as well as the ubiquitin E3 ligase Hel2 as neighbors of Asc1. We observed Asc1-dependency of *hr40S* localization of mRNA-binding proteins and the Ubp3 co-factor Bre5. Hel2 and Ubp3-Bre5 are described to balance the mono-ubiquitination of Rps3 (uS3) during ribosome quality control. Here, we show that the absence of Asc1 resulted in massive exposure and accessibility of the C-terminal tail of its ribosomal neighbor Rps3 (uS3). Asc1 and some of its direct neighbors together might form a ribosomal decision tree that is tightly connected to close-by signaling modules.

## 1. Introduction

Connectivity of membrane-separated cellular processes in eukaryotes depends on signal transduction to bridge intra-cellular distances and organelle barriers. Ribosomes localize for site-specific and process-bound protein biosynthesis. They are connected to local signaling entities, for example, membrane receptors, kinase modules, and/or sources of secondary messengers, to facilitate timely reactions to stimuli. Conversely, they have to communicate their own status, like translational arrest or ribosome stalling, to initiate processes such as ribosome clearance, ribosome preservation, or ribophagy. Moreover, they must forward ribosome status messages, for example, into the nucleus for the adaptation of genome activities.

The Asc1 protein of *Saccharomyces cerevisiae* is a highly conserved eukaryotic seven-bladed β-propeller of the WD40-repeat protein family and named RACK1 in plants and mammals. It is a canonical ribosomal protein and gets incorporated late into the 40S ribosome during cytosolic maturation [1,2]. Cellular depletion of this protein in *S. cerevisiae* (*asc1*^−^ cells) causes massive proteome and transcriptome changes [3,4]. RACK1 gene knock-down or mRNA/protein depletion in higher eukaryotes is lethal during early embryogenesis, and medical studies have provided ample evidence that aberrant RACK1 expression in mammals coincides with different types of cancer [5,6,7,8,9]. Yeast *asc1*^−^ cells proliferate well in culture, although they display a tremendously altered phosphoproteome [4,10]. Furthermore, they show several phenotypical traits, for example, loss of dimorphism (haploid adhesive and diploid pseudohyphal growth), increased sensitivity to osmotic constraint and heat, slow-growth on non-fermentable carbon sources, increased sensitivity to translation inhibitors and genotoxic compounds, and cell cycle delay [3,4,11,12,13,14].

Asc1/RACK1 resides at the head region of the 40S ribosomal subunit (*hr40S*), close to the mRNA entry and exit tunnels of initiating 40S and translating/stalled 80S ribosomes [15,16]. Remodeling of protein composition and arrangement within this microenvironment is expected to account for ribosome-activity adaptations to meet cellular needs. Conversely, even the ribosomal translation status itself is expected to influence protein assembly at the *hr40S*. The analysis of the remodeling of cellular microenvironments is frequently done with co-immunoprecipitation and tag-affinity capture. These techniques rely on high binding affinities of proteins within complexes that have to endure cell lysis and the subsequent affinity purification procedure. They sometimes suffer from artificial protein–protein binding within cell extracts that have lost the cellular order. New approaches employ the covalent labeling of proteins of a microenvironment within living cells. This enables the subsequent capture and identification of adjacent proteins irrespectively from their behavior upon and after cell lysis. Our previous microenvironment analysis using a Biotin IDentification approach (BioID) [17] for *S. cerevisiae* combined with quantitative LC-MS analysis provided a first Asc1-proxiOME: beyond expected ribosomal Asc1-neighboring proteins, we identified, for example, the ubiquitin E3 ligase Hel2, the deubiquitinating complex Ubp3-Bre5, and various mRNA-binding proteins, amongst them, the mRNA-exonuclease Xrn1 [18]. Also, Def1, a protein known to act in concert with the Elongin-Cullin E3 ligase complex to trigger proteasomal RNA polymerase II subunit degradation in the nucleus, was found to be in the proximity of Asc1 [18,19].

RACK1/Asc1, and its neighbor Hel2, function in the ribosome-associated quality control (RQC) and no-go-decay (NGD) systems that are required for the degradation of aberrant polypeptides and mRNA clearance upon abnormal translation arrest. Ribosome stalling during translation elongation is caused by various translation compromising factors, such as rare codons within the translated mRNA (reviewed in [20]). Increasing data imply a RACK1/Asc1 function within early RQC to initiate the degradation of nascent aberrant peptide chains of stalled 80S ribosomes [21]. Also, the endonucleolytic cleavage of stalled non-stop mRNAs (non-stop decay, NSD) depends on RACK1/Asc1 [22]. Splitting apart the 40S and 60S-nascent-peptide-chain subunits of stalled ribosomes depends on the ubiquitination of 40S proteins through Hel2 (ortholog of human ZNF598) and is followed by Rqc2-and Hel2-dependent CATylation of split 60S-nascent-peptide-chains [21,23,24]. The C-terminal tail of the 40S ribosomal protein S3 (Rps3/uS3) binds Asc1, thereby linking it to the mRNA guiding tunnel and the mRNA decoding area in 80S ribosomes [15]. Physical interaction between mRNA and Rps3 at the entry tunnel of the small ribosomal subunit promotes NGD of aberrant mRNAs [25,26]. Mono-ubiquitination within the C-terminal tail of Rps3 is critical for RQC and correlates with translational stalling. This, in turn, depends on the balanced activities of the E3 ubiquitin ligase Hel2 and of the deubiquitinase complex Ubp3-Bre5 (ortholog of human USP10-G3BP1) [27,28]. Ubp3-Bre5 also controls ribophagy, the ultimate route towards bulk degradation of dispensable ribosomes [29]. We showed previously that phosphorylation of Bre5 at serine 282 is Asc1-dependent [10].

Here, we intended to broaden the current knowledge of the *hr40S* microenvironment through affinity capture from the Asc1-adjacent perspective of Rps2 (uS5). Our study focuses on the analysis of the Asc1-dependency of the *hr40S* composition. We went for a comparative BioID experiment expressing the Rps2-BirA* fusion protein in both wild-type *ASC1* and *asc1*^−^ cells. The localization of Bre5 at *hr40S* turned out to depend on Asc1. By contrast, Vps30 is captured upon Rps2-BirA* biotinylation only in the absence of Asc1. Vps30 (ortholog of human Beclin-1) is a subunit of the phosphatidylinositol (PtdIns) 3-kinase complexes I and II, which are involved in autophagosome formation and vacuolar protein-sorting [30]. Furthermore, the Rps2-BioID experiments confirmed the localization of Def1 to the *hr40S*. A picture emerges with Asc1/RACK1 acting as a context-bound signal-coupling factor that coordinates ribosome activity and homeostasis. During this process, it might couple ribosomal status changes with nuclear genome activities via ubiquitin- and phosphorylation-mediated protein translocation. The *hr40S* might serve as a wharf for protein arrival and departure. Twenty-five years after launching mass spectrometry-based proteomics, proxiOME approaches, like BioID and the recently developed context-specific variant of split-BioID [31], are new and promising proteomics techniques to study meaningful cellular sub-proteomes. They might have the power to illuminate otherwise invisible molecular ships passing in the night (expression with reference to Toby J. Gibsons article *RACK1 research—ships passing in the night?* [32]).

## 2. Materials and Methods

### 2.1. Plasmid Construction

All plasmids used in this study are listed in Table 1. The RPS2-birA* plasmid pME4799 is equivalent to the ASC1-birA* bearing plasmid pME4478 and was generated using the GeneArt^®^ Seamless Cloning and Assembly Enzyme Mix Kit (#A14606, Thermo Fisher Scientific).

### 2.2. Yeast Strains and Growth Conditions

Yeast strains used in this study are either of the Σ1278b or S288c background and are listed in Table 2. Yeasts cells were grown in liquid yeast nitrogen base medium (YNB; 0.15% YNB, 0.5% ammonium sulfate, 2% glucose) with additional supplements as required: L-arginine (20 mg/l), L-lysine HCl (30 mg/L), L-tryptophan (20 mg/L), uracil (20 mg/L), L-leucine 30 mg/L), L-valine 150 mg/L), L-isoleucine (30 mg/L). To obtain the methionine prototrophic strain RH3793, the wild-type *MET15* gene was reintroduced into the genome of the Bre5-GFP expressing strain from Invitrogen. This was done to avoid suppression of the *MET15* promoter that regulates the expression of RPS2-birA* and birA*. The *ASC1* gene was deleted in strain RH3793 by replacing it with *LEU2* (derived from plasmid pUG73) resulting in strain RH3794. Deletion cassettes for the replacement of *BRE5* and *UBP3* with the *kanMX4* resistance marker were amplified from genomic DNA isolated from the respective Euroscarf deletion strains. The deletion cassettes were transformed into the *ASC1* wild-type strain (RH2817) and the *asc1*^−^ strain (RH3510), resulting in strains RH3789-3792.

### 2.3. Western Blot Analysis

Cell lysis was performed as described by Kushnirov (2000) [36]. For each sample, an equal number of cells (OD_600_ of 2.5, calculated according to the OD_600_ of the corresponding culture) were harvested by centrifugation, washed with water, and resuspended in 200 µL 0.1 M NaOH. Samples were incubated for 5 min at room temperature and centrifuged at 4000 rpm for 50 s. The supernatant was discarded, and 50 µL of 1:4 diluted loading dye (see below for composition) was added. Samples were heated for 3 min at 95 °C, centrifuged at 16,200× *g* for 5 min, and 6 µL of the supernatant were subjected to SDS-PAGE followed by the transfer onto nitrocellulose membrane. Proteins on the membrane were visualized by staining with Ponceau Red (0.2% PonceauS, 3% trichloroacetic acid). After blocking, the membranes were incubated either with polyclonal rabbit anti-Asc1p [10], or polyclonal rabbit anti-Rps3 (provided by Heike Krebber), or monoclonal mouse anti-GFP (B-2, sc-9996, Santa Cruz Biotechnology) followed by incubation with the respective peroxidase-coupled secondary antibody. For the detection of biotinylated proteins, membranes were blocked overnight with 1% BSA in phosphate buffered saline (PBS, 8 mM Na_2_HPO_4_, 2 mM NaH_2_PO_4_, 150 mM NaCl). Pierce^TM^ High Sensitivity Streptavidin-HRP (#21130, Thermo Fisher Scientific, diluted 1:2000 in the blocking buffer with 0.1% Tween 20) was added for 1 h to the membranes. Chemiluminescent signals were detected using the FUSION-SL-4 (Peqlab).

### 2.4. BioID Experiments

Yeast cells were cultivated in 200 mL YNB medium to mid-log phase. The media contained 10μM D-biotin and differentially labeled lysine and arginine for SILAC. The following amino acids were used for differential labeling: ^13^C_6_-L-arginine HCl, ^13^C_6_^15^N_4_-L-arginine HCl, 4,4,5,5-D_4_-L-lysine HCl, ^13^C_6_-L-lysine HCl. Cell pellets were washed twice with 10 mL of washing buffer 1 (10 mM HEPES, pH 7.9, 10 mM KCl, 1.5 mM MgCl_2_) and mixed in a 1:1:1 ratio of cells according to the OD_600_ of the cultures. Cells were lysed in breaking buffer (wash buffer with 1 cOmplete^TM^ protease inhibitor cocktail tablet (Merck) per 50 mL, 0.5 mM DTT, and 0.5 mM PMSF) using glass beads. SDS was added to a final w/v of 4% for the denaturation of proteins. Enrichment of biotinylated proteins was performed according to the protocol described in Opitz et al. (2017) [18]. Strep-Tactin^®^ Sepharose^®^ gravity flow columns with 0.2 mL instead of 1 mL bed volume were used (#2-1202-550, IBA GmbH). The cell extract was applied to the column, and the column was washed with 10 mL 1x washing buffer 2 (#2-1003-100, IBA GmbH) containing 0.4% SDS. The biotinylated proteins were eluted with 600 µL 1x washing buffer containing 10 mM D-biotin. Proteins were precipitated according to Wessel and Flügge (1984) [37] by chloroform-methanol extraction. The cell pellet was dissolved in 20 µL 8 M urea/2 M thiourea and subjected to SDS-PAGE. Gel lanes were cut into 8 pieces each, and proteins were digested in-gel with trypsin [38]. Peptides were desalted with C18 (3M) stop and go extraction (stage) tips [39,40]. Peptides were dried and then dissolved in a 20 µL sample buffer (2% acetonitrile, 0.1% formic acid) for the subsequent LC-MS analysis. Strep-Tactin^®^XT spin columns (#2-4150-025, IBA GmbH) were used to enrich biotinylated peptides according to the manufacturer’s instructions. Peptides were eluted using three times the 150 µL elution buffer with 50 mM biotin. Peptides were purified again using stage tips and subjected to LC-MS analysis.

For the Western blot analysis of input samples and column flow-through, 3x loading dye (0.25 M Tris-HCl pH 6.8, 30% glycerol, 15% β-mercaptoethanol, 7% SDS, 0.3% bromphenol blue) was added to the respective fractions, and the samples were heated at 65 °C for 10 min. Equal volumes of the input and flow-through samples were subjected to SDS-PAGE.

For BioID experiments with subsequent Western blot analysis, cells were cultivated in 150 mL YNB medium to mid-log phase in the presence of 10 μM D-biotin. The preparation of cell extracts and enrichment of biotinylated proteins were performed as already described, except that 100 µL Strep-Tactin^®^ Sepharose^®^ Slurry (50 µL bed volume, #2-1201-002, IBA GmbH) was used instead of gravity flow columns. Cell lysates were incubated with the equilibrated beads on a rotating wheel for 30 min. Beads were washed three times with 800 µL washing buffer containing 0.4% SDS. To elute proteins from the beads, 75 µL 2x loading dye were added, and samples were heated for 3 min at 95 °C.

### 2.5. Liquid Chromatography-Mass Spectrometry (LC-MS) Analysis

Liquid chromatography systems (UltiMate 3000 RSLCnano system) coupled to either an Orbitrap Velos Pro hybrid ion trap-Orbitrap or a Q Exactive HF mass spectrometer (all Thermo Fisher Scientific) were used for peptide analysis. Peptides in 4 µL sample solution were trapped and washed on an Acclaim PepMap 100 precolumn (C18; 100 µm, 2 cm, 3 µm, 100 Å; Thermo Fisher Scientific) at a flow rate of 25 µL/min for 6 min in 100% solvent A (98% water, 2% acetonitrile, 0.07% trifluoroacetic acid). Analytical peptide separation by reverse-phase chromatography was performed on an Acclaim PepMap RSLC column (C18; 75 µm, 50 cm, 3 µm, 100 Å; Thermo Fisher Scientific), running a gradient from 98% solvent A (0.1% formic acid) and 2% solvent B (80% acetonitrile, 0.1% formic acid) to 7% solvent B within 3 min, to 32% solvent B within 92 min, and to 65% solvent B within the next 26 min, at a flow rate of 300 nL/min (solvents and acids were from Fisher Chemical). For the chromatography of the Q Exactive HF coupled system, a similar gradient was used with minor changes. Eluting peptides were ionized online by nanoelectrospray ionization (nESI) using a Nanospray Flex ion source (Thermo Fisher Scientific) at 1.5 kV (Orbitrap Velos Pro) and 1.6 kV (Q Exactive HF) and were continuously transferred into the mass spectrometer. Full scans within the mass range of 300 to 1850 m/z were taken within the Orbitrap-FT analyzer at a resolution of 60,000 with parallel data-dependent top 15 MS2 collision induced dissociation (CID) fragmentation with the LTQ Velos Pro linear ion trap. For the Q Exactive HF, the mass range of the full scans was 300 to 1650 m/z and data-dependent top 10 MS2 fragmentation with higher-energy collisional dissociation (HCD) fragmentation was performed. The software XCalibur 2.2 (Thermo Fisher Scientific) was used for LC-MS method programming and data acquisition. MS/MS2 data were searched against an *S. cerevisiae*-specific protein database (UniProt Proteome ID UP000002311) using the software MaxQuant 1.6.0.16 [41]. The digestion mode was trypsin/P, and the maximum number of missed cleavage sites was set to three. Arg6, Arg10, Lys4, and Lys8 were defined as the medium and heavy labels in accordance with the amino acids used for cultivation of the cells. Carbamidomethylation at cysteine was set as a fixed modification, and oxidation at methionine and biotinylation of lysine were set as variable modifications. Mass tolerances of precursors and fragment ions were 4.5 ppm and 0.5 Da (CID) or 20 ppm (HCD), respectively. Re-quantification was enabled. At a minimum, two ratio counts were required for protein quantification using unique and razor peptides. False discovery rates were calculated using the revert decoy mode, and the threshold for peptide sequence matches as well as protein identifications was 0.01. MaxQuant output data were further evaluated using the Perseus software 1.6.0.7 [42].

### 2.6. Phenotypic Growth Tests

Yeast cells were grown to mid-log phase in YNB medium and diluted to an OD_600_ of 0.1. Ten-fold dilutions were prepared, and 10 or 20 µL of each dilution were dropped on yeast extract peptone dextrose (YEPD) or YNB plates, respectively. The plates were incubated for 3 days at 25 °C, 30 °C, or 37 °C. Additional compounds were added, as indicated in Figure 4. To test for haploid adhesive growth, cells were patched on 10 mM 3-amino-1,2,4-triazole (3-AT) containing YNB plates and incubated for 3 days at 30 °C. Plates were washed under a constant stream of water to test the ability of the yeast cells to adhere to the surface of the agar plate.

### 2.7. Accession Number

Data are available via ProteomeXchange with identifier PXD015611 [43,44].

## 3. Results

### 3.1. Expression of Rps2-BirA* in S. cerevisiae Strains of the Σ1278b Genetic Background

**Figure 1:**
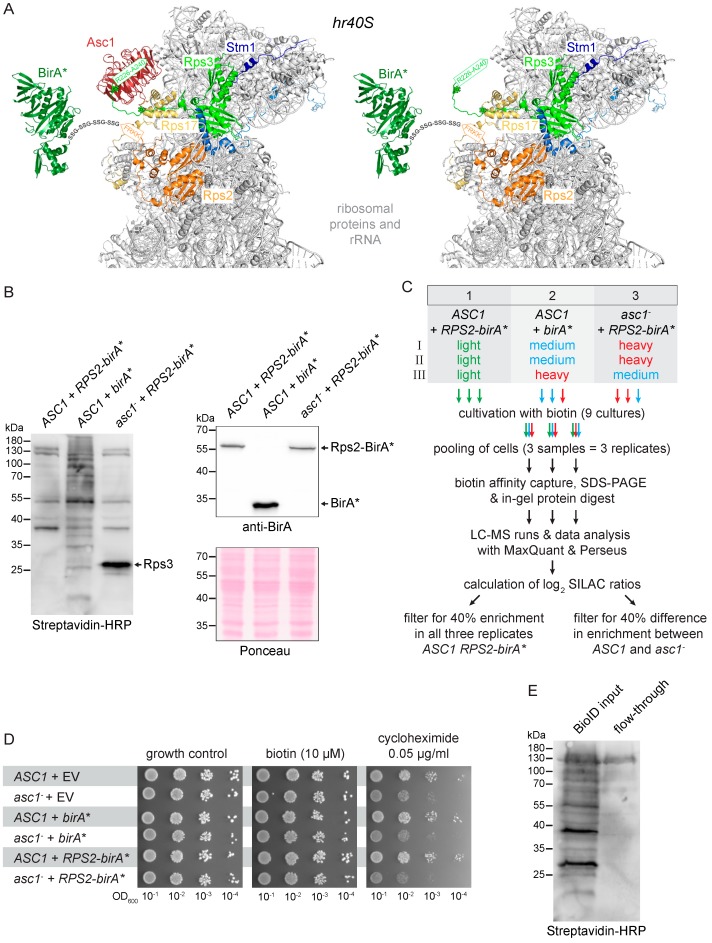
Set-up of the Rps2-BioID analysis. (**A**) Rps2-BirA* at the *hr40S* in the presence and absence of Asc1 (red). The BirA* protein (dark green) is fused to the C-terminus of Rps2 (uS5, orange) with a linker sequence consisting of four repeats of Gly-Ser-Ser (GSS). The last four amino acids of Rps2 (in orange letters) were structurally not resolved and are indicated within an orange arrow. Ribosomal proteins captured with Rps2-BirA* are highlighted in different colors: Rps17 (eS17) yellow, Rps3 (uS3) light green, Stm1 blue. Biotinylated lysine residues K212 and K223 of Rps3 are indicated with green asterisks (the green arrow indicates the last 15 C-terminal amino acids of Rps3 that were structurally not resolved). The PyMOL Molecular Graphics System software was used to generate the figures from crystal structure data of the *S. cerevisiae* 80S ribosome and the *E. coli* BirA protein derived from the PDB entries 4V88 [15] and 1BIB [45], respectively. (**B**) An aliquot of each SILAC-labeled culture was taken prior to cell pooling for cell lysis. Extracts were applied to SDS-PAGE and proteins blotted to a membrane. Streptavidin-HRP was used to detect biotinylated proteins (left), and a BirA-specific antibody was applied to detect the BirA* and Rps2-BirA* proteins (right). The birA* and RPS2-birA* alleles were expressed from extrachromosomal high copy number plasmids in the *ASC1* wild-type and *asc1*^−^ background as indicated. Ponceau staining of the proteins on the membrane served as the loading control. Results are shown representatively for one of three replicates. (**C**) Strategy and workflow of the Biotin IDentification (BioID) experiment. Stable isotope labeling with amino acids in cell (SILAC) amino acids were used for a triple-labeling approach with three replicates (I-III), including a medium to heavy label swap. For detailed information about mass-spectrometry data acquisition (XCalibur) and analysis (MaxQuant and Perseus), see Materials and Methods and Table 3. (**D**) Drop dilution assays (successive 10-fold dilutions) in the presence of biotin or cycloheximide to test whether the additional expression of BirA* or Rps2-BirA* impairs colony growth. Strains transformed with the empty vector (EV) were used for growth controls. (**E**) Binding of biotinylated proteins on Strep-Tactin gravity flow columns. Equal volumes of the cell lysate (BioID input, applied on the Strep-Tactin column) and the flow-through were subjected to SDS-PAGE and proteins blotted to a membrane followed by detection of biotinylated proteins using streptavidin-HRP.

**Figure 2:**
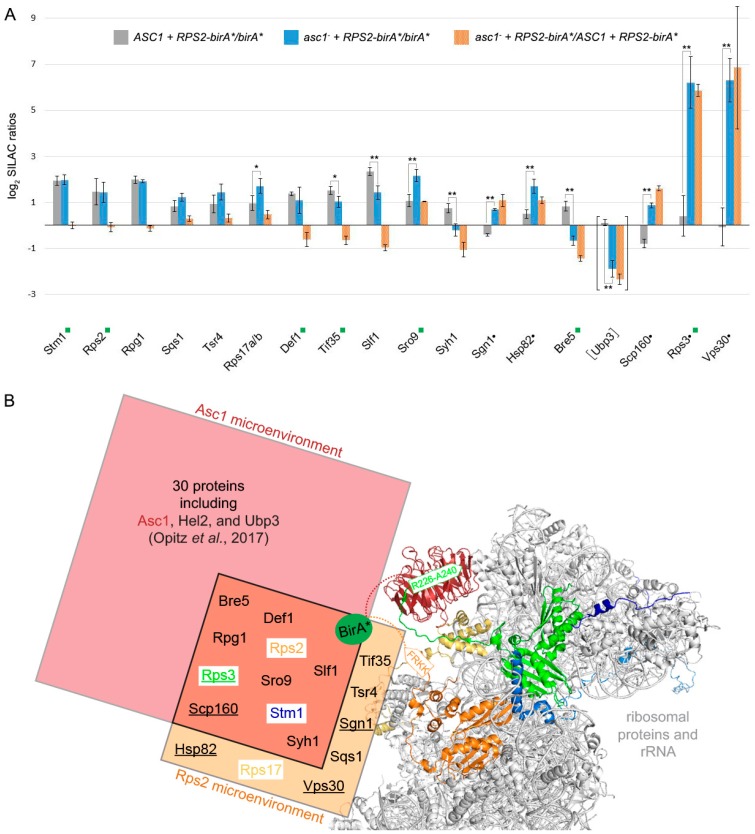
BioID protein capture after Rps2-BirA*-labeling in the presence or absence of Asc1. (**A**) All columns represent the average of MaxQuan*t* normalized log_2_ SILAC ratios of three biological replicates and are provided with the respective standard deviations. Proteins depicted in the graph were enriched with normalized log_2_ SILAC ratios for ASC1 RPS2-birA*/birA* greater than or equal to 0.485 (matching a minimum enrichment of approximately 40%) from either *ASC1* wild-type cells (gray columns) and/or from *asc1*^−^ cells (blue columns). Black dots next to protein names indicate proteins that were exclusively enriched from the *asc1*^−^ cells. Orange columns represent the respective fold-changes of the SILAC protein quantification values between *ASC1* wild-type and *asc1*^−^ cells, indicating the degree of Asc1-dependency in their Rps2 proximal localization. The proteins are ordered from left to right according to increasing absolute differences in enrichment between *ASC1* wild-type and *asc1*^−^ cells (orange columns). A two-sample t-test on the ASC1 RPS2-birA*/birA* versus asc1^−^ RPS2-birA*/birA* log_2_ SILAC ratios was performed, and significant changes were indicated with one or two asterisks (*p*-value threshold 0.05 and 0.01, respectively). A green rectangle next to the protein name indicates the identification of a biotinylated peptide for the respective protein. Although the data for Ubp3 did not pass the described filtering, they are provided for the discussion (see main text). The graph is based on the data in Table S1. (**B**) Comparison of the Asc1 and Rps2 microenvironments captured with BioID: The *hr40S* is illustrated as in Figure 1A. The biotin ligase BirA* (green sphere) was fused via a linker sequence to the C-termini of either Asc1 or Rps2 (indicated with dashed lines in red and orange, respectively). The proteinaceous microenvironment of Asc1 [18] is indicated with a red rectangle and that of Rps2 with an orange one. Proteins identified in the neighborhood of both proteins are listed in the overlap of both rectangles and are supposed to belong to a common microenvironment. Proteins only identified in the absence of Asc1 within the Rps2 microenvironment are underlined. Proximity between Asc1 or Rps2 to their neighboring proteins might also take place apart from a mature 40S ribosomal subunit. The PyMOL Molecular Graphics System software was used to generate the figure from crystal structure data of the *S. cerevisiae* 80S ribosome (PDB entry: 4V88) [15].

**Figure 3:**
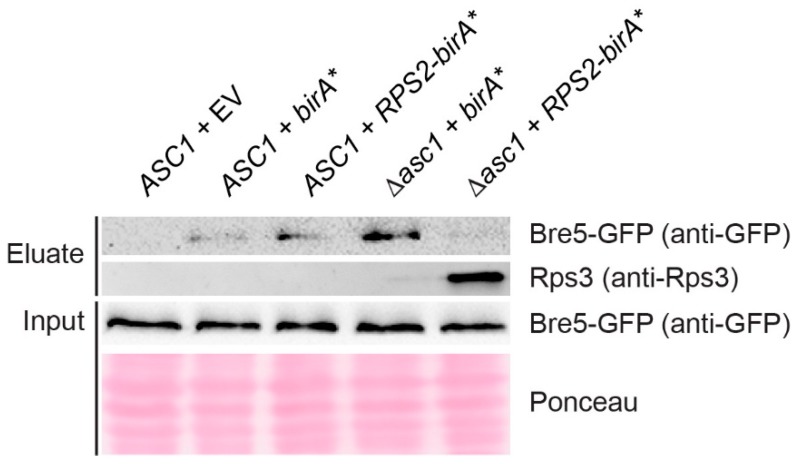
Immuno-detection of Bre5 and Rps3 captured with a small-scale Rps2-BirA* BioID experiment. Rps2-BirA* BioID capture was performed in a small-scale format (see Materials and Methods for details) using strains expressing GFP-tagged Bre5. BirA* and Rps2-BirA* were expressed from high copy number plasmids in *ASC1* wild-type and *asc1*^−^ strains as indicated, and the *ASC1* wild-type strain was additionally transformed with the empty vector (EV) as a control. Western blot experiments were performed with a GFP-antibody and an Rps3-antibody to detect protein enrichment in eluate fractions. Bre5-GFP was also detected in the total cell extracts (input). Ponceau staining of proteins served as a loading control for the input.

Our original inventory analysis of the Asc1-proxiOME using BioID revealed some expected neighboring proteins, for example, known adjacent ribosomal proteins and subunits of eIF3, as well as some unexpected ones, such as the deubiquitinase complex Ubp3-Bre5 and the RNA polymerase II degradation factor Def1. Using BioID experiments from the perspective of the Asc1-adjacent Rps2 protein, we intended first to confirm the presence of those proteins that were not expected at the head region of the 40S subunit (*hr40S*), and second, to study the putative Asc1-dependency of their *hr40S* localization. For the second aim, we compared Rps2-BioID captures from wild-type *ASC1* and *asc1*^−^ cells. This should reveal the impact of Asc1 on *hr40S* composition and/or dynamics. Rps2 was chosen as a new perspective for in vivo labeling of *hr40S* proteins with biotin because its C-terminus extends to this region (Figure 1A) [15]. Additionally, Rps2 was specifically biotin-labeled and captured upon Asc1-BirA* expression [18]. Also, the functionality of previously published C-terminal Rps2 fusion proteins (e.g., with GFP) encouraged us in the choice of Rps2 as BioID bait [29]. Similar to our Asc1-BioID experiments, we expressed an Rps2-BirA* fusion protein from a high-copy number plasmid in wild-type *RPS2 ASC1* as well as in *RPS2 asc1*^−^ cells (Figure 1A). *asc1*^−^ refers to an allele encoding an *asc1* mRNA with a stop codon in exon 1, abrogating translation; the pre-mRNA still contains the *SNR24* gene within its intron [3]; in the following text, we refer to this allele as *ASC1* deletion as it does not produce any Asc1 protein. As a negative control, we also transformed *RPS2 ASC1* cells with only the unfused birA* allele on a high-copy number plasmid, giving rise to Rps2-independent background biotin-labeling activity. BirA* and Rps2-BirA* expression and the stability of the fusion protein in both strain backgrounds were confirmed by Western blot experiments using an anti-BirA antibody (Figure 1B). Overall protein biotinylation in cells grown overnight in the presence of biotin was analyzed for the respective strains using horseradish peroxidase (HRP)-coupled streptavidin on Western-blot membranes (Figure 1B). The expression of mere BirA* gave rise to significantly increased background biotinylation in comparison to Rps2-BirA* expression. Rps2-BirA* expression in *asc1*^−^ cells generated a very strong additional biotinylation signal at a molecular weight of approximately 26 kDa derived from Rps3 (as shown later, Figure 2A and Figure 3).

We next confirmed that Rps2-BirA* expression did not compromise the general growth of wild-type *ASC1* or *asc1*^−^ cells. Without further stress applied, the cell growth of all tested strains was similar in the absence as well as the presence of external biotin (Figure 1D). As expected, *asc1*^−^ cells were sensitive to the translation inhibitor cycloheximide [10]. Expression of Rps2-BirA* as an additional Rps2 copy did not change cycloheximide sensitivity. Comparative quantitative Rps2-BioID capture experiments with strains expressing Rps2-BirA* in both *ASC1* and *asc1*^−^ cells (strains 1 and 2) and mere BirA* in *ASC1* cells (strain 3) were performed using stable isotope labeling with amino acids in cell culture (SILAC; Figure 1C). Cellular proteins of these three yeast strains were differentially labeled with mass-specific variants of the SILAC amino acids lysine and arginine (e.g., strain 1 with light, strain 2 with medium, and strain 3 with heavy isotope amino acids incorporated) during overnight growth in the presence of 10 μM biotin. An equal number of cells from all three cultures were combined to yield one common cell pool for protein isolation and the biotin capture procedure. The quantitative Rps2-BioID experiment was performed in triplicate and was done as described previously for the Asc1-BioID in Opitz et al. (2017) [18]. The procedure applied is summarized in the Materials and Methods section. The only difference was the use of Strep-Tactin columns with 0.2 mL instead of 1 mL bed volume. As shown in Figure 1E, the smaller bed volume bound biotinylated proteins from cell extracts efficiently.

We identified 431 proteins with SILAC ratios for all three biological replicates. The ratios normalized by MaxQuant were used for data analysis. A detailed workflow for the data analysis in Perseus is provided in Table 3. Proteins that were enriched from RPS2-birA* expressing cells by more than 40% compared to the control in all three replicates were considered as Rps2 neighbors (normalized log_2_ SILAC ratios RPS2-birA*/birA* greater than or equal to 0.485, Figure 2A). Four proteins, Stm1, Rpg1, Sqs1, and Tsr4 were identified as Rps2 proximal proteins independent of the presence of Asc1. Seven additional proteins, Rps17 (eS17), Def1, Tif35, Slf1, Sro9, Syh1, and Bre5 were also identified in the Rps2 microenvironment but showed Asc1-dependency in their enrichment. Five proteins, Sgn1, Hsp82, Scp160, Rps3, and Vps30, were only captured with Rps2-BioID in the absence of Asc1. The identification of known neighbors of Rps2 at ribosomes, such as Rps17, Stm1, and the translation initiation factor 3 subunits a/Rpg1 and g/Tif35 [15,46,47], proves that the Rps2-BioID method is a reliable approach to uncover proximities. Furthermore, the protein Tsr4 was just recently described as a chaperone for Rps2 and interacts with its eukaryotic-specific N-terminal region [48,49]. In total, 9 of the 16 identified Rps2-neighbors were also captured with Asc1-BirA* [18], showing that we were able to monitor a common microenvironment of both proteins at the *hr40S* (Figure 2B).

### 3.2. Rps2-BirA* Specifically Biotinylates Def1 and Stm1

Using Rps2-BirA*, we identified Def1 as a protein whose localization at the *hr40S* depends on Asc1 (Figure 2A). Upon transcriptional arrest, Def1 induces ubiquitination of the largest subunit of the stalled RNA polymerase II complex, Rbp1. A prerequisite for the transfer of Def1 from the cytoplasm to the nucleus is a substantial proteasome-mediated protein trimming through chopping off more than 200 amino acids of its Q-rich C-terminus [19]. Our previous Asc1-BioID experiment indicated the presence of the non-processed full-length Def1 version in the proximity of Asc1 [18]. At that time, we could not discriminate whether the two proteins were within proximity at or off the *hr40S*. The rediscovery of Def1 in the Rps2-BioID experiment here (Figure 2A) gives further evidence that Def1 actually resides at the *hr40S* close to Asc1 prior to its transfer into the nucleus. We previously showed that phosphorylation of Def1 at threonine 258 and serine 260 is Asc1-dependent [10]. Phosphorylation might play a role in this specific targeting of Def1.

Stm1 previously appeared as an Asc1 proximal protein [18], and it is an interactor of the translation elongation factors eEF3/Yef3 [50] and eEF2/Eft1 [51]. It resides as a putative subunit clamping factor in stalled ribosomes during glucose starvation [15], acts as a ribosome preservation factor during stationary growth [50], and has reappeared here in the Rps2-BioID experiment. The protein is homologous to the putative mammalian ortholog SERBP1 that has been described to interact with RACK1 in HeLa cells [52]. Localization of Stm1 at the *hr40S* does not depend on Asc1 (Figure 2A). However, its remodeling into the ribosome clamping position upon starvation might be mediated by Asc1.

### 3.3. Asc1 Inversely Affects Rps2-BirA*-Mediated Biotinylation of the mRNA-Binding Paralogs Sro9 and Slf1

Sro9 and Slf1 are two other proteins whose Rps2-BirA*-dependent biotinylation varied significantly in the *asc1*^−^ compared to the *ASC1* strain (Figure 2A). They are paralogous atypical-La motif-containing mRNA-binding proteins, and Sro9 was shown to shuttle between the nucleus and the cytoplasm in an mRNA-dependent manner [53]. Sro9 is also part of the heat-shock protein-containing HMC complex repressing the transcriptional activator heme activator protein 1 (Hap1) in the absence of heme. In the cytoplasm, Sro9 associates with translating ribosomes [54]. Our previous Asc1-BioID experiment suggested its precise location to be proximal to Asc1 [18]. The Rps2-BioID experiment now confirmed the *hr40S* as the site for Sro9 localization (Figure 2A). The absence of Asc1 at the *hr40S* even increased the capture of Sro9 as well as that of Scp160 and Sgn1 (two further mRNA-binding proteins). In contrast, the enrichment of the Sro9 paralogous protein Slf1, a key activator of translation during the oxidative stress response [55], decreased in the absence of Asc1. Asc1 seems to balance the localization of various mRNA-binding proteins at the *hr40S*.

### 3.4. Asc1 Inversely Affects Rps2-BirA*-Mediated Biotin-Enrichment of Bre5 on the One Hand and Rps3 and Vps30 on the Other Hand

Ubp3 and Bre5 together form a deubiquitinase complex and have both been identified as Asc1-proximal with our original Asc1-BioID experiment [18]. Bre5 reappeared in the Rps2-BioID experiment confirming its localization to the *hr40S* (Figure 2A). The capture of biotinylated Bre5 upon Rps2-BirA* expression is significantly reduced in the absence of Asc1; increased background biotinylation of Bre5 in merely BirA* expressing cells (negative control) even led to negative Rps2-BirA*-mediated enrichment values after normalization. (This is also the case for Syh1, a paralog of Smy2 that participates in COPII vesicle formation, Figure 2A) [56]. The increased biotinylation of Bre5 in the negative control was confirmed by Western blot experiments with eluates of the Rps2-BioID experiment of yeast strains expressing Bre5-GFP by use of an anti-GFP antibody. Bre5-GFP background biotinylation upon BirA* expression significantly increases in the absence of Asc1, whereas Bre5-GFP biotinylation upon Rps2-BirA* expression fades in *asc1*^−^ cells (Figure 3). We showed previously that phosphorylation of Bre5 at serine 282 ceases upon depletion of Asc1 [10]. Asc1-dependent Bre5 phosphorylation might take place in a regulatory context of ribosomal subunit ubiquitination through Ubp3 to remodel ribosomes according to cellular signals. Ubp3 was not enriched from *ASC1* wild-type cells in comparison to the birA* control, possibly caused by even stronger background biotinylation. However, it was significantly less captured from *asc1*^−^ cells, indicating similar Asc1-dependent binding to *hr40S* as Bre5.

In contrast to the decreasing biotinylation and BioID capture of Bre5 in *asc1*^−^ cells, the ribosomal protein Rps3 gets increasingly BioID-captured in the absence of Asc1 upon cellular Rps2-BirA* expression (Figure 2A). This finding is supported by Western blot experiments using an anti-Rps3 antibody (Figure 3). According to the crystal structure of the 80S ribosome of Ben-Shem et al. (2011) [15], Rps3 physically contacts Asc1 with its C-terminal tail. The removal of Asc1 seems to expose parts of Rps3, rendering its tail prone to Rps2-BirA*-mediated biotinylation (see also Figure 1A, right-hand side).

Vps30, a subunit of the phosphatidylinositol 3-kinase (PI3K) complexes I and II [30], was not captured upon Rps2-BirA* expression from *ASC1* wild-type cells at all. It was, however, strongly enriched from *asc1*^−^ cells (Figure 2A). The strong Vps30 enrichment at the *hr40S* equals that of Rps3 from *asc1*^−^ cells. Future studies might reveal whether the exposure of the C-terminal Rps3 tail is required for Vps30 association to the *hr40S* and whether other subunits of the PI3K complexes locate to ribosomes to induce signaling events.

### 3.5. ASC1 Genetically Interacts with UBP3/BRE5 to Protect Cells Against Heat, Starvation, and Translational Stress

**Figure 4:**
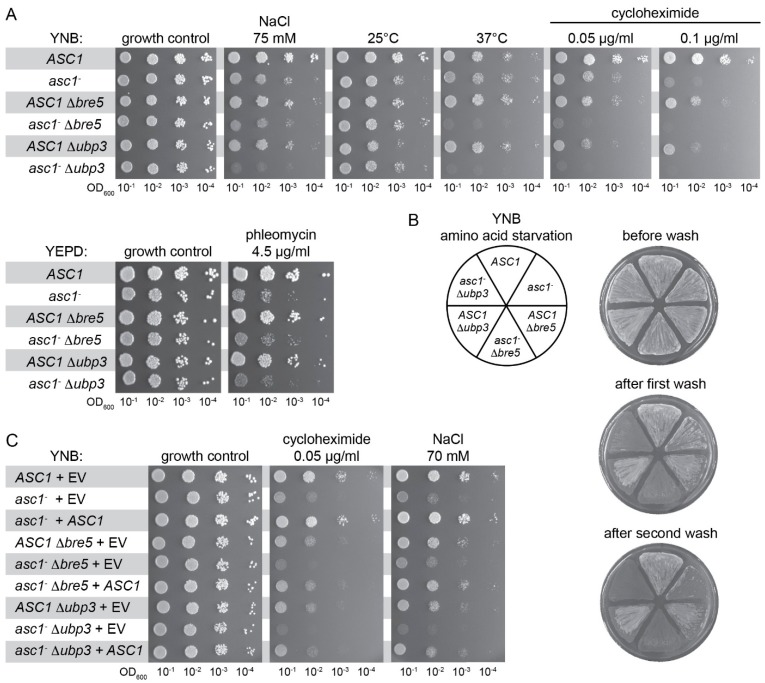
Genetic interaction between *ASC1* and *BRE5*/*UBP3***.** *S. cerevisiae ASC1*, *BRE5*, and *UBP3* single and double deletion strains were phenotypically characterized. (**A**) Drop dilution assays were performed to test for sensitivity of the strains to cycloheximide, phleomycin, as well as to hyperosmotic stress (NaCl), cold stress (25 °C), and heat stress (37 °C). (**B**) Cells were grown on 3-AT containing plates to analyze adhesive growth. The plate was photographed before and after treatment with a constant stream of water (shorter and longer wash). (**C**) The *asc1*^−^ strain and the double deletion strains *asc1*^−^ ∆*bre5* and *asc1*^−^ ∆*ubp3* were complemented with plasmid-borne *ASC1.* As controls, the wild-type strain and all single and double deletion strains were transformed with the empty vector (EV). Drop dilution growth assays were performed to test for sensitivity to cycloheximide and hyperosmotic stress (NaCl).

The increasing evidence for a functional interaction between Asc1 and the deubiquitinase Ubp3-Bre5 complex prompted us to analyze putative genetic interactions of their encoding genes. In the absence of cellular stress, single or double deletions of the genes *ASC1*, *UBP3*, and *BRE5* (*asc1*^−^, Δ*bre5*, Δ*ubp3*, *asc1*^−^ Δ*bre5*, and *asc1*^−^ Δ*ubp3* strains) do not compromise colony growth (Figure 4A, growth control in comparison to the respective wild-type strain). Growth at hyperosmotic conditions (75 mM NaCl), at elevated temperatures (37 °C instead of 30 °C), or in the presence of the translation inhibitor cycloheximide (0.05 and 0.10 μg/mL), is mildly compromised for cells with the single deletions *asc1*^−^, Δ*bre5*, and Δ*ubp3*. The double deletions *asc1*^−^ Δ*bre5* and *asc1*^−^ Δ*ubp3*, however, lead to synergistic growth defects. Growth is almost abolished at elevated temperatures and in the presence of the translation inhibitor cycloheximide. In contrast to the growth phenotype on 37 °C, double mutation strains grew slightly better than the single deletion strains at lower temperatures (25 °C).

Bilsland and colleagues described the sensitivity of Δ*bre5* and Δ*ubp3* cells to the DNA damaging agent phleomycin [57]. Growth in the presence of 4.5 μg/mL phleomycin is significantly impaired for *asc1*^−^ cells and to a lesser extent for Δ*bre5* and Δ*ubp3* cells. Double deletion strains *asc1*^−^ Δ*bre5* and *asc1*^−^ Δ*ubp3* do not show synergistic growth impairment and resemble the *asc1*^−^ phenotype upon phleomycin treatment (Figure 4A). Adhesive cell growth of wild-type *S. cerevisiae* cells of the genetic Σ1278b background is generally caused by nutrient depletion, for example, starvation through prolonged growth on solid medium or in the presence of starvation-inducing agents (e.g., the histidine analog 3-aminotriazole causing amino acid starvation; shown in Figure 4B). Patches of wild-type cells remain stuck on the surface after exposing them to a stream of water (Figure 4B, *ASC1* pie slice). Also, Δ*bre5* and Δ*ubp3* cells stick to the surface (Figure 4B, *ASC1* Δ*bre5* and *ASC1* Δ*ubp3* pie slices). Patches of *asc1*^−^ Δ*bre5* and *asc1*^−^ Δ*ubp3* cells, however, are easily washed off the plates after only a short exposure time to a water stream, while the patch of *asc1*^−^ cells still remains stuck at the surface (Figure 4B, first wash). A more intensive washing also removed the patches of *asc1*^−^ cells from the surface in contrast to the *ASC1* wild-type cells that still stick (Figure 4B, second wash). Transformation of the different *asc1*^−^ strains (*asc1*^−^, *asc1*^−^ Δ*bre5*, and *asc1*^−^ Δ*ubp3*) with the *ASC1* gene on a centromere plasmid complemented the Asc1-dependent growth phenotypes as expected (Figure 4C, for growth in the presence of the translation inhibitor cycloheximide, 0.05 μg/mL, and growth at hyperosmotic conditions, 70 mM NaCl). In summary, the phenotypic characterization of *ASC1*, *UBP3*, and *BRE5* single and double deletion strains also revealed genetic interactions that give further evidence for a cooperation of the encoded proteins at the *hr40S*.

## 4. Discussion

Protein phosphorylation and ubiquitination steer cellular decisions according to numerous intra- and extra-cellular signals. They cause remodeling of protein–protein interactions and, thus, of protein complexes and microenvironments. We have analyzed the head region of the 40S ribosomal subunit (*hr40S*) and consider protein remodeling there both as a prerequisite for and a consequence of ribosome activity and homeostasis. Initiation of mRNA translation, as well as ribosome stalling, might influence the protein composition and arrangement at *hr40S*. Now, we have analyzed this microenvironment with BioID capture experiments from two adjacent perspectives, namely the Asc1 and the Rps2 perspectives. As expected, the two neighboring viewpoints yield a similar but not identical repertoire of proximal proteins (Figure 2B). Variations in the lengths of the linker sequence between bait and biotin ligase and the occupation of different adjacent perspectives will contribute to the spatial resolution and orientation of proteins identified with BioID within a microenvironment.

The accumulation of nucleocytoplasmic-shuttling mRNA-binding proteins and of subunits of the translation initiation complex eIF3 (a/Rpg1 and g/Tif35) at the *hr40S* indicates that substantial cargo reception and translation initiation processes take place at this site. We provide evidence that yRACK1/Asc1 affects the positioning of the mRNA binding proteins Scp160, Sgn1, and the paralogous proteins Sro9 and Slf1. Scp160 is a well-established Asc1 interacting protein, and it was repeatedly shown that ribosome-association of Scp160 is reduced in the absence of Asc1 [58,59]. However, we made the converse observation and captured more Scp160 from the ribosomal Rps2-perspective when Asc1 was absent. An explanation might be that Asc1 shields Scp160 from biotinylation through Rps2-BirA*. Alternatively, the assays to evaluate the ribosome association of Scp160 might reflect the strength of its binding rather than its overall localization at ribosomes. The position of Scp160 at the ribosome in Asc1-deficient cells might be shifted, rendering Scp160 prone to dissociation from the ribosome during ultracentrifugation. In contrast, BioID identifies proximities without necessarily reflecting the affinity of interactions.

The mRNA-binding protein Sro9 has been described as a subunit of the heat-shock 70 protein (Hsp70)-containing complex (HMC). This complex represses the transcription factor heme activator protein 1 (Hap1) in the absence of heme [60]. Hap1, in turn, mediates oxygen sensing and heme signaling. There is a genetic link between *ASC1* and oxygen-related response mechanisms: *ASC1* was originally discovered in a genetic screen by Verdière and colleagues as a gene in which inactivation allowed for the growth of otherwise inviable heme-depleted *hap1* mutant cells [61]. In line with this, we observed an impact of Asc1 on the ability of cells to switch from fermentative to respiratory growth [3]. In the presence of heme, enhanced association of Hsp90 with the HMC complex causes Hap1 activation [60]. Coincidently, with the increased Rps2-BioID capture of Sro9 from *asc1*^−^ cells, a similar increase of the Hsp90 family chaperone Hsp82 was observed. The Sro9 paralog Slf1 has also been described as a key regulator of the oxidative stress response [55]. The results presented here indicate that cooperation of Asc1 with Sro9, Slf1, and heat shock proteins at the *hr40S* might account for the genetic link between *ASC1* and *HAP1*. In mammalian cells, oxygen sensing and the transcriptional response of oxygen-dependent genes are mediated through HIF1-α, a transcription activator protein whose activity and stability are also controlled through a phosphorylation/ubiquitination-mediated interplay with heat-shock proteins and RACK1 [62]. We consider the ribosomal head region as a decisive site for Asc1/RACK1-mediated signal transfer to regulate the stability and nuclear transport of Hap1/HIF1-α.

Def1 is a CUE domain-containing protein that resides predominantly in the cytoplasm. However, it translocates into the nucleus to designate mRNA polymerase II for ubiquitination and proteasome-mediated degradation [19]. Prior to its nuclear translocation, full length Def1 obviously resides at the *hr40S* within the cytoplasm as observed with our Asc1-BioID experiments and now confirmed with the Rps2-BioID. The positioning of Def1 at the *hr40S* and its phosphorylation at threonine 258 and serine 260 are Asc1-dependent (results here and Schmitt et al., 2017 [10], respectively), and might together be important for the proteasome-mediated cropping of its Q-rich C-terminus that precedes its nuclear transport [19].

The deubiquitinase Ubp3 and its cofactor Bre5 (USP10 and G3BP1 in mammalian cells, respectively) are important factors controlling ribophagy and mitophagy [29,63,64] and were previously found to be Asc1-proximal [18]. Here, we observed the Asc1-dependent localization of Bre5 at the *hr40S,* as examined from the Rps2-BirA* viewpoint. We assume that the same is true for Ubp3, but strong background biotinylation in *ASC1* wild-type cells expressing BirA* alone (see biotinylation within the negative control in Figure 1B, middle lane) did not lead to Rps2-BirA*-mediated Ubp3 enrichment ratios from *ASC1* wild-type cells. Nevertheless, there was significant Ubp3 deprivation in *asc1*^−^ cells at the *hr40S* (Figure 2A), leading us to cautiously claim Asc1-dependency for the Ubp3-localization as well. Beyond Asc1-dependent localization of Ubp3-Bre5 to the ribosomal *hr40S*, we showed evidence for the genetic interaction of Asc1 with Ubp3-Bre5 under various stress conditions. The Ubp3-Bre5 complex plays an essential role in the assembly of cytoplasmatic non-membranous ribonucleoprotein (RNP) stress granules that encompass stalled translation pre-initiation complexes [65]. A balanced mono-ubiquitination and deubiquitination of the ribosomal protein S3 (Rps3), mediated by Ubp3 and the E3 ubiquitin ligase Hel2 (ZNF598 in mammalian cells), has been demonstrated to be important for ribosome-associated quality control and autophagy processes [27]. Previously, we found Hel2 proximal to Asc1 when investigated from the Asc1-BirA* perspective [18]. However, it was not visible from the Rps2-BirA* viewpoint in this study. Rps3 gets ubiquitylated at K212 and K223 within its C-terminal tail that is physically shielded through and bound by Asc1 (Figure 1A). Also, acetylation and succinylation of K212 of Rps3 and phosphorylation of close-by sites have been described [66,67,68]. Both lysine residues are unshielded in *asc1*^−^ cells leading to lysine biotinylation through Rps2-BirA* and, accordingly, to strong Rps3 biotin capture. Interaction between mRNA and Rps3 at the ribosomal mRNA entry tunnel at the 40S subunit supports both translation initiation accuracy [69] and the endonucleolytic cleavage of aberrant mRNAs during no-go decay (NGD) [25]. Ribosome collision after stalling is critical for triggering RQC activities, for example, endonucleolytic mRNA cleavage [26]. In mammalian systems, stalling is recognized by the Hel2 ortholog ZNF598 [70]. In *S. cerevisiae*, Hel2 was shown to bind to mRNAs at translating 40S ribosomal subunits preferentially near stop codons through the interaction with 18S rRNA and Asc1 [71]. Asc1 and Rps3 also act together in nonfunctional 18S rRNA decay (18S NRD) [72]. A Cryo-EM structure of two collided 80S ribosomes revealed that the *hr40S*-Asc1 microenvironment is the main physical contact site, with both Asc1 molecules directly interacting [73]. Collision might couple two 80S ribosomes via Asc1 homodimerization that, in turn, displace the C-terminal tail of Rps3 that binds Asc1 at its dimerization interface [15,74]. A displaced C-terminal Rps3 arm could then be prone to protein modifications that might be important to trigger subsequent steps in ribosome conversion or conservation. The ribosome preservation factor, Stm1, cogent BioID candidate in both Asc1- and Rps2-BioID experiments, could then be inserted into the non-translating ribosomes as a surrogate for degraded mRNA to function as a subunit clamping factor that conserves ribosomes during early starvation periods.

Severe nutrient starvation causes ribophagy, a mechanism for bulk degradation of non-translating ribosomes within the vacuole [29]. Autophagosome formation involves the generation of phosphatidylinositol 3-phosphate that is generated by phosphatidylinositol 3-kinases (PI3Ks). Our BioID experiments revealed that an integral component of the PI3K complexes I and II, Vps30, occupies a position at the *hr40S* proximal to Rps2 in the absence of Asc1. Mammalian RACK1 was described to promote the formation of a PI3K complex comprising the ortholog of Vps30, Beclin-1, and, thus, autophagosome induction [75]. Future studies will address the question of whether there are physiological conditions that induce a displacement of Asc1 in favor of Vps30 localization at the *hr40S*. This might target or activate PI3K complexes to initiate ribophagy.

There is considerable heterogeneity of ribosomes and probably of the regulatory head region of the 40S subunit as well. Depending on the cellular signals, ribosomes are supposed to be more generally or context-specifically regulated in terms of activity and homeostasis. Our Asc1- and Rps2-BioID experiments do not yet resolve the diversity at the *hr40S*. However, they provide a first insight into this complex microenvironment. Improved faster and smaller BioID versions will contribute to further temporally resolved protein remodeling within microenvironments [76,77]. Emerging split-BioID approaches will allow the detection of common microenvironments of proximal proteins. This method combines two chosen proximal proteins fused to inactive halves of a biotin ligase, which will only become active upon protein interaction [31]. Such context-specific split-BioID experiments, in combination with genetically engineered protein isoforms and/or specific signals/growth conditions, are planned to dissect the diversity of proteins identified and to break down the analyses of the dynamic *hr40S* microenvironments to specific sets of ribosomes.

## Figures and Tables

**Table 1:** Plasmids used in this study.

Plasmid Name	Description	Reference
pME2783	pRS416*MET25* with *MET25Prom*, *CYC1Term*, *URA3, CEN/ARS*	[33]
pME2787	pRS426*MET25* with *MET25Prom, CYC1Term*, *URA3*, 2 μm	[33]
pME4364	*CYC1Term*, *URA3, CEN/ARS, ASC1* with its native promoter (+500 bp)	[10]
pME4480	*MET25Prom*, *CYC1Term*, *URA3*, 2 μm, birA*	[18]
pME4478	*MET25Prom*, *CYC1Term*, *URA3*, 2 μm, ASC1-birA*	[18]
pME4799	*MET25Prom*, *CYC1Term*, *URA3*, 2 μm, RPS2-birA*	This study
pUG73	*LEU2* deletion cassette	[34]

**Table 2:** *Saccharomyces cerevisiae* strains used in this study.

Strain Name	Description	Background	Reference
RH2817	*MATα*, *ura3-52*, *trp1::hisG*	Σ1278b	[4]
RH3510	*MATα*, *ura3-52*, *trp1::hisG*, *asc1-loxP SNR24*	Σ1278b	[3]
RH3493	*MAT*α, *ura3-52, trp1::hisG,* Δ*arg4::loxP,* Δ*lys1::loxP*	Σ1278b	[10]
RH3520	*MATα*, *ura3-52*, *trp1::hisG*, *asc1-loxP SNR24*, ∆*arg4::loxP*, ∆*lys1::loxP*	Σ1278b	[10]
Y06078	*MATa; ura3Δ0; leu2Δ0; his3Δ1; met15Δ0; BRE5::kanMX4*	S288c	Euroscarf
Y06148	*MATa; ura3Δ0; leu2Δ0; his3Δ1; met15Δ0; UBP3::kanMX4*	S288c	Euroscarf
RH3789	*MATα*, *ura3-52*, *trp1::hisG*, ∆*bre5::kanMX4*	Σ1278b	This study
RH3790	*MATα*, *ura3-52*, *trp1::hisG*, *asc1-loxP SNR24,* ∆*bre5::kanMX4*	Σ1278b	This study
RH3791	*MATα*, *ura3-52*, *trp1::hisG*, ∆*ubp3::kanMX4*	Σ1278b	This study
RH3792	*MATα*, *ura3-52*, *trp1::hisG*, *asc1-loxP SNR24,* ∆*ubp3::kanMX4*	Σ1278b	This study
BRE5-GFP	*MATa*, *ura3Δ0*, *leu2Δ0*, *his3Δ1*, *met15Δ0*, *BRE5-GFP_HIS3MX6*	S288c	Invitrogen, [35]
RH3793	*MATa*, *ura3Δ0*, *leu2Δ0*, *his3Δ1*, *MET15*, *BRE5-GFP_HIS3MX6*	S288c	This study
RH3794	*MATa*, *ura3Δ0*, *leu2Δ0*, *his3Δ1*, *MET15*, *BRE5-GFP_HIS3MX6,* ∆*asc1::LEU2*	S288c	This study

**Table 3:** Overview of MaxQuant output data evaluation with Perseus. The table lists the main steps of the Perseus analysis.

No.	Command	Description
1	Generic matrix upload	proteinGroups.txt normalized ratios, etc.
2.1	Filter rows based on categorical columns	Remove rows with “+” in reverse column
2.2		Remove rows with “+” in potential contaminant column
2.3		Remove rows with “+” in only identified by site column
3	Transform	Inverse ratios (1/x), ratios are reported as follows: ASC1 RPS2-birA*/birA* asc1^−^ RPS2-birA*/birA* asc1^−^ RPS2-birA*/ASC1 RPS2-birA*
4	Transform	log_2_(x)
5	Filter rows based on valid values	9 valid values in total, Reduce matrix (431 proteins remained)
6.1	Categorical annotation rows	Group biological replicates 1: ASC1 RPS2-birA*/birA*
6.2		2: asc1^−^ RPS2-birA*/birA*
6.3		3: asc1^−^ RPS2-birA*/ASC1 RPS2-birA*
6.4		4: ASC1 RPS2-birA*/birA* and asc1^−^ RPS2-birA*/birA* (for two sample t-test)
7.1 7.2	Two-sample tests	Select the two groups in 4 (see 6.4), *p*-value threshold: 0.05 *p*-value threshold: 0.01
Filter for proteins enriched from ASC1 RPS2-birA*		
8	Filter rows based on valid values	3 valid values in group 1 greater than or equal to 0.485 (approximately 40% enrichment), reduce matrix
9	Average groups	Calculate mean and standard deviation
Filter for proteins enriched from asc1^−^ RPS2-birA* but not from ASC1 RPS2-birA*		
10	Filter rows based on valid values	3 valid values in group 3 outside -0.485 and 0.485 Reduce matrix
11	Filter rows based on valid values	3 valid values in group 2 greater than or equal to 0.485, Reduce matrix
12	Average groups	Calculate mean and standard deviation

## Supplementary Materials

The following are available online at https://www.mdpi.com/2073-4409/8/11/1384/s1, Table S1: LC-MS identification and quantification data for proteins identified in proximity of Rps2-BirA*. Data correspond to Figure 2A.
